# Flag leaf chlorophyll content of wheat: genetic dissection, candidate genes prediction and its relationship with other agronomic traits

**DOI:** 10.3389/fpls.2026.1938691

**Published:** 2026-09-04

**Authors:** Jiajun Liu, Nana Qin, Defu Wang, Jingtao Liu, Xiaorong Liu, Lan Qi, Yuyin Li, Hongbin Peng, Huaping Tang, Jian Ma, Qiang Yang

**Affiliations:** 1 Dazhou Key Laboratory of Agricultural Resources Development and Ecological Conservation in Daba Mountain/School of Ecotourism, Sichuan University of Arts and Science, Dazhou, China; 2 Cereal and Oil Crops Research Institute, Dazhou Academy of Agricultural Sciences, Dazhou, China; 3 State Key Laboratory of Crop Gene Exploration and Utilization in Southwest China/Triticeae Research Institute, Sichuan Agricultural University, Chengdu, China; 4 Institute of Quality Standard and Testing Technology Research, Sichuan Academy of Agricultural Sciences, Chengdu, China

**Keywords:** wheat, flag leaf chlorophyll content, QTL mapping, candidate genes, pleiotropic

## Abstract

Chlorophyll is the primary pigment responsible for capturing light energy and driving photosynthetic conversion. As the key photosynthetic organ during grain filling, the flag leaf plays a critical role in carbon assimilation, consequently, its chlorophyll content (FLC) represents a promising target for enhancing wheat yield and ensuring food security. Dissecting the genetic basis underlying FLC and elucidating its relationships with other agronomic traits are essential for accelerating molecular breeding aiming at improving photosynthetic efficiency and yield in wheat varieties. In this study, a major and environmentally stable quantitative trait locus (QTL) for FLC was detected using a population of 197 recombinant inbred lines (RILs). This QTL, designated *QFLC.scwl-4B*, was consistently mapped to chromosome arm 4BS across multiple environments, explaining 9.36%–31.82% of the phenotypic variance (PVE). The effect of *QFLC.scwl-4B* was further validated in an additional RIL population under diverse environmental conditions. Correlation and effect analyses revealed that *QFLC.scwl-4B* exhibits pleiotropic, leading to increased FLC, spikelet number per spike (SNS), and flag leaf thickness (FLT), alongside reduced productive tiller number (PTN). Furthermore, a putative candidate gene for *QFLC.scwl-4B*, *TraesCS4B03G0098300*, was identified as an ortholog of gene in BBX family, which is known to repress chlorophyll degradation and senescence, and likely involved in chlorophyll synthesis. Further, the promoter region of *TraesCS4B03G0098300* harbors a single nucleotide polymorphism (G/C) between the two parents. Collectively, these findings provide valuable QTL resources and candidate gene targets for marker-assisted selection in breeding programs aiming at developing wheat varieties with high photosynthetic efficiency and yield.

## 1 Introduction

Wheat (*Triticum aestivum* L.) is a globally important staple grain crop, and its yield is critical to food security (Feng et al., 2025; Han et al., 2026). During grain filling, the flag leaf serves as the primary photosynthetic organ, providing the majority of carbohydrates required for grain development (Wang et al., 2025). Consequently, its photosynthetic efficiency directly influences grain yield. Chlorophyll, the principal pigment for responsible light energy capture and photosynthetic conversion (Chaudhary et al., 2022), is a key determinant of leaf photosynthetic capacity. Therefore, enhancing flag leaf chlorophyll content (FLC) represents a promising strategy for achieving high and stable wheat yields.

Numerous studies have demonstrated a significant positive correlation between FLC and yield-related traits, including the net photosynthetic rate, thousand grain weight (TGW), and biomass accumulation (Avenson et al., 2005; Chen et al., 2025). Moreover, FLC serves as an important indicator of leaf senescence and nitrogen remobilization efficiency (Xiong et al., 2015), both of which are closely associated with grain filling and the final yield. Elevated FLC during the grain-filling period can delay senescence, prolong the duration of photosynthetic, and promote the translocation of assimilates to developing grains (Cui et al., 2026; Ibrahim et al., 2025). Therefore, dissecting the genetic loci and functional genes underlying FLC is of paramount importance for breeding wheat varieties with improved photosynthetic performance and enhanced yield potential.

As a complex quantitative trait, FLC is governed by multiple minor-effect genes and exhibits complex genotype-by-environment interactions (Yang et al., 2007; Zhang et al., 2009a). Owing to advances in molecular marker technology, hundreds of quantitative trait loci (QTLs) controlling chlorophyll content have been identified in wheat. For example, *qChla5D* and *qChlb5D* on chromosome 5D accounted for 12.95% and 23.29% of the phenotypic variance (PVE), respectively (Zhang et al., 2009b). A locus on chromosome 4D was associated with chlorophyll content (Li et al., 2010), and *QTc.wwc-1B-S11* on chromosome 1B explained 10.09% of the PVE (Ilyas et al., 2014). Under different water conditions, a total of 50 QTLs for soil plant analysis development (SPAD) value were detected (Yang et al., 2016b), while 157 FLC QTLs were identified under varying phosphorus conditions, among which six were considered as major QTLs (Yang et al., 2022). Additionally, three QTLs (*Qchl.saw-2D.1*, *Qchl.saw-4D.2* and *Qchl.saw-6A*) were detected across multiple environments, with PVE values range from 4.10% to 26.07% (Yang et al., 2023).

In addition, several QTLs and genes associated with stay-green traits have been successively reported. For instance, Shi et al. (2017) detected 28 QTLs for chlorophyll content under diverse water regimes, with PVE ranging from 4.60% to 21.40%. Two major QTLs, *QSg.sau-2B.1* and *QSg.sau-6A.2*, were detected in at least three environments, explaining 6.38%–23.85% and 14.02%–17.48% of the PVE, respectively (Ren et al., 2022). Furthermore, several stay-green genes, including *Tackx4* (Chang et al., 2015), *Tabas1-B1* (Zhu et al., 2016), *els1* (Li et al., 2018), *TaPPH-7A* (Wang et al., 2019) and *Els2* (Wang et al., 2020) have been identified in diverse wheat germplasm through map-based and homologous cloning approaches.

Although extensive efforts have been made to map loci regulating FLC, most reported QTLs have been identified using low-density markers in a single environment or under stress condition. Environmentally stable and major QTLs applicable to breeding programs remain to be identified and characterized. Hence, there is an urgent need to screen a broader range of wheat germplasm to identify novel and major QTLs with stable expression and major effects on FLC. In this study, two recombinant inbred line (RIL) populations were employed to detect and validate such loci for FLC across multiple environments. The primary objectives were thus: (1) to identify and validate major and stable QTLs for FLC; (2) to investigate the correlations between FLC and agronomic, grain-related, and root system traits, as well as the genetic effect of the major QTLs on these traits; and (3) to predict candidate genes underlying the major QTLs. These findings provide a theoretical foundation and genetic resources for marker-assisted selection of wheat varieties with desirable FLC levels.

## 2 Materials and methods

### 2.1 Plant materials

In this study, a previously reported RIL population (2CN), comprising 197 F_7_ lines derived from a cross between 20828 and Chuannong 16 (CN16), was used for QTL mapping (Liu et al., 2018). The wheat line 20828 (G214-5/3/Chuanyu19//Lang9247/50788) exhibits high spikelet number per spike (SNS) and strong resistance to stripe rust (Ma et al., 2019a, Ma et al., 2019b). CN16 (Chuanyu12/87-429), a nationally approved cultivar, possesses a higher productive tiller number (PTN) and high TGW (Liu et al., 2018; Ma et al., 2019c). To verify the genetic effect of the major QTL, an additional RIL population (2SY) consisting of 126 F_7_ lines derived from a cross between 20828 and SY95–71 was also employed (Liu et al., 2020).

### 2.2 Field experiments

The 2CN population was planted at Beidou (BD, 107°45′E, 31°15′N) in 2024 and 2025, at Micheng (MC, 107°19′E, 31°18′N) in 2024, and at Kaijiang (KJ, 107°50′E, 31°13′N) in 2025. The 2SY population was planted at MC in 2024, at KJ in 2025, and at BD in 2025. In each environment, the populations were arranged in a randomized complete block design with a single replicate. All field trials were conducted using single-row plots with a row length of 1.5 m and 15 seeds per row. The within-row spacing was 0.1 m, and the between-row spacing was 0.3 m. Plots were randomized independently randomized within each environment, and crop management followed standard local practices for wheat production.

### 2.3 Phenotypic evaluation

SPAD provides a direct estimate of the relative chlorophyll content in leaves (Netto et al., 2005). FLC of the main stem was determined using a chlorophyll meter (SD-1CK, Beijing Times Maker Technology Co., Ltd.) between 7:00 to 10:00 a.m. after flowering (Yang et al., 2016a). Five plants per line were sampled, and the average value was calculated for subsequent analysis. The FLC phenotypes of the 2CN and 2SY populations were evaluated across four (2025BD, 2026BD, 2025MC, 2026KJ) and three (2025MC, 2026KJ, 2026BD) environments, respectively, with the year in each environment-location label indicating the season of trait evaluation.

Additionally, best linear unbiased prediction (BLUP) datasets of other traits in the 2CN population were obtained from our previously published studies, including: plant height (PH) and spike length (SL; Li et al., 2019), flower period (FP) and spikelet number per spike (SNS; Ma et al., 2019a), productive tiller number (PTN; Liu et al., 2018), spike extension length (SEL; Li et al., 2020a), flag leaf length and width (FLL, FLW; Ma et al., 2020), flag leaf thickness (FLT; Liu et al., 2026), TGW (Ma et al., 2019c), grain length and width (GL, GW; Ma et al., 2019c), grain number per spike (GNPS; Ma et al., 2019a), grain weight per spike (GWPS; Ma et al., 2020), and root system traits (Li et al., 2020b). The root system traits encompassed total root length (TRL), root surface area (RSA), root volume (RV), root average diameter (AD), maximum root length (MRL), number of root tips (RTN), shoot dry weight (SDW), and root dry weight (RDW).

### 2.4 QTL analysis

A high-density genetic map of the 2CN population based on the Wheat 55K SNP array in our previous study was used (Liu et al., 2018). QTL analysis was performed using the inclusive composite interval mapping (ICIM) method implemented in the biparental population (BIP) module of QTL IciMapping v4.2 (Meng et al., 2015), with parameters set as follows: Step = 1.0 cM, PIN = 0.001, and logarithm of odds (LOD) threshold = 2.50. QTL-by-environment interactions were identified using the multi-environmental trial (MET-ADD) module with the same parameter settings. Epistatic interactions were detected using the epistatic (EPI) module in QTL IciMapping, with parameters set to LOD = 5.0, PIN = 0.001, and Step = 1.0 cM. According to the user manual of QTL IciMapping v4.2, missing phenotypic data are replaced with -100 when performing QTL calculations. QTL were named based on the guidelines for gene nomenclature in wheat (Boden et al., 2023). “FLC” and “scwl” denote “Flag leaf chlorophyll” and “Sichuan University of Arts and Sciences”, respectively.

### 2.5 Validation of the major QTL

To validate the genetic effects of the major QTL, we evaluated FLC in the 2SY population across multiple environments. Genotyping data for markers *AX-109526283* and *AX-109469073* in this population were retrieved from our previous study (Liu et al., 2020). Based on these marker genotypes, the 2SY population was divided into two groups: lines with homozygous alleles from the parent 20828 and homozygous alleles from SY95-71. Subsequently, phenotypic differences in FLC between the two groups were compared using Student’s *t*-test (*P* < 0.05).

### 2.6 Assessing the impact of 1BL/1RS translocation on FLC

CN16 is a nationally approved wheat cultivar carrying the 1BL/1RS translocation (Qi et al., 2016). Given that the 1RS- or 1BS-specific markers exhibited both co-segregation and distorted segregation in the 2CN RIL population (Liu et al., 2018), we estimated the potential effect of this translocation on FLC. Lines carrying either 1RS (n = 33) or 1BS (n = 138), as previously reported by Liu et al. (2018), were compared for FLC using Student’s *t*-test (*P* < 0.05) in SPSS. Furthermore, Student’s *t*-test (*P* < 0.05) was also used to compare the FLC between the 1RS (n = 17) and 1BS (n = 70) lines that lacked the major FLC locus (**Supplementary Table 1**).

### 2.7 Prediction of candidate genes

In this study, the sequences of flanking markers linked to the major QTL were retrieved from our previous study (Liu et al., 2018). These flanking marker sequences were aligned to the Chinese Spring reference genome (CS v2.1; Zhu et al., 2021) to determine the physical interval of the major QTL. Gene annotations and expression patterns of the predicted genes were obtained from WheatOmics (https://wheatomics.sdau.edu.cn/). In addition, the genomic sequences of the parental lines 20828 and CN16 were acquired from previous studies (Liu et al., 2025). Subsequently, the coding sequence (CDS) and promoter sequence of *TraesCS4B03G0098300* were extracted from these genomic sequences and subjected to sequence alignment.

### 2.8 Comparison with previously reported QTL for FLC

To determine whether the QTL identified in this study is a novel locus, we compared its physical interval with those of previously reported FLC-related QTL or genes by anchoring the corresponding flanking marker sequences to the CS v2.1 genome assembly.

### 2.9 Statistical analysis

Phenotypic variation, frequency distribution, and Pearson’s correlations coefficients across environments were analyzed using the SPSS 24 program (IBM, Armonk, NY, USA; https://www.ibm.com/products/spss-statistics). Student’s *t*-test (*P* < 0.05) was performed to detect significant differences in a given trait between parental lines, or between different RIL groups. SAS v8.0 (SAS Institute, Cary, NC, USA; https://www.sas.com/) was employed to estimate the BLUP and broad-sense heritability (*H^2^*) of FLC across environments. All figures were generated using Origin Pro 2021 v9.8.3 (https://www.originlab.com/).

## 3 Results

### 3.1 Phenotypic analyses

**Figure 1:**
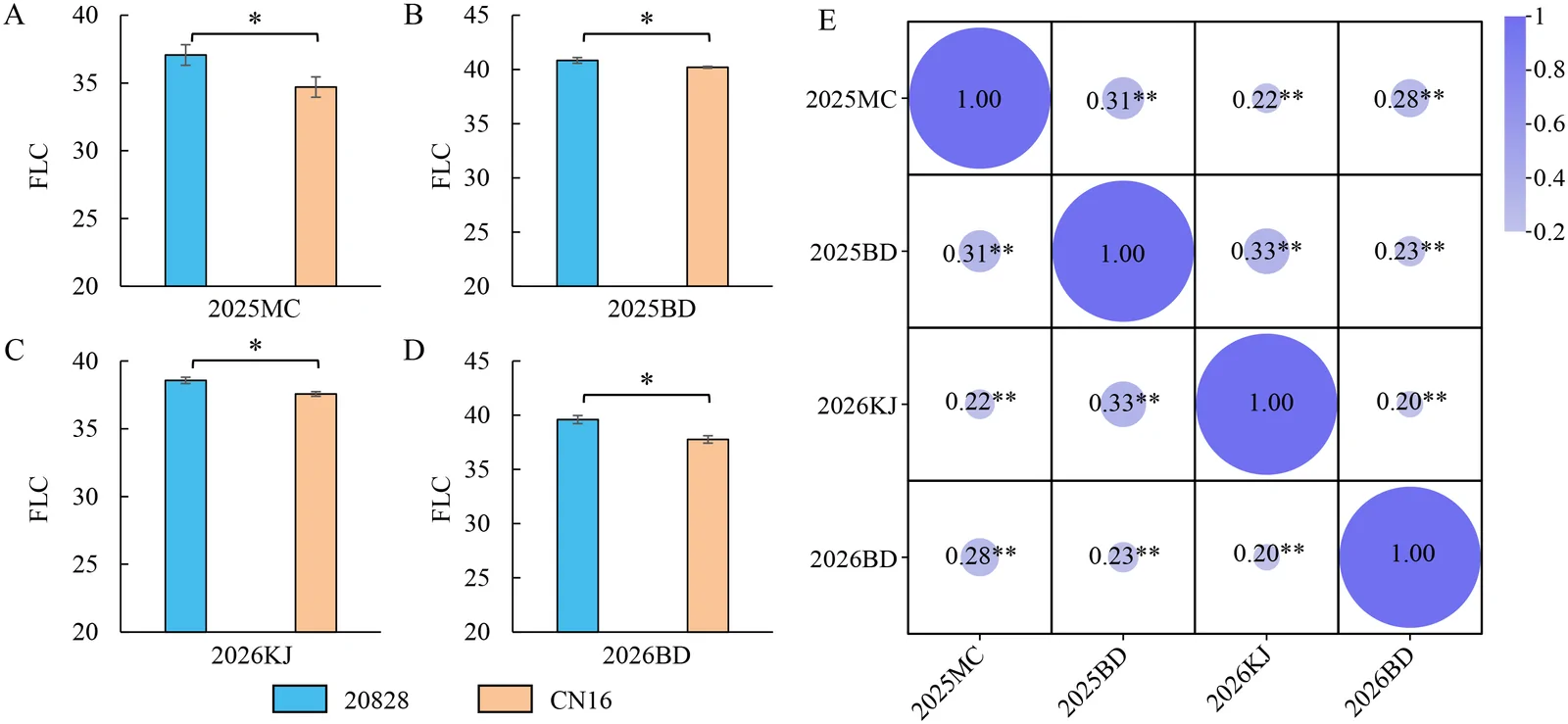
Phenotypes analysis of the parents **(A–D)** and correlation analysis of the test environment **(E)**. FLC, Flag leaf chlorophyll content; 2025MC, Micheng in 2025; 2025BD, Beidou in 2025; 2026KJ, Kaijiang in 2026; 2026BD, Beidou in 2026. * represents significant differences at the 0.05 levels, the *P*-values.

Significant differences in FLC between the parents 20828 and CN16 were observed across all four environments (*P* < 0.05; **Figures 1A–D**). In the 2CN population, FLC values ranged from 24.77 to 46.67, with coefficients of variation (*CV*) ranged from 2.06% to 7.77% (**Table 1**). The *H^2^* of FLC in the 2CN populations were 0.48 (**Table 1**), indicating FLC was influenced by both genetic and environmental factors. The frequency distribution of FLC exhibited an approximately normal and continuous distributions (**Supplementary Figure 1**), suggesting that FLC is a typical quantitative trait and thus suitable for QTL analysis. Additionally, significant positive correlations for FLC were detected among the four environments (*P* < 0.01; **Figure 1E**).

**Table 1:** Phenotypic variation of flag leaf chlorophyll content (FLC) in 2CN population grown in different environments.

Environment	2CN RIL population ^a^			
	Range	Mean ± SD	*CV*	*H^2^* ^b^
2025MC	33.80–43.13	37.87 ± 1.72	4.55%	0.48
2025BD	33.07–46.67	40.63 ± 2.43	5.99%	
2026KJ	31.83–41.30	35.35 ± 1.60	4.51%	
2026BD	24.77–40.40	35.47 ± 2.75	7.77%	
BLUP	35.33–39.41	37.31 ± 0.77	2.06%	

RIL, Recombinant inbred line; SD, Standard deviation; *CV*, Coefficient of variation; *H^2^*, Broad-sense heritability; 2025MC, Micheng in 2025; BD, Beidou in 2025; 2026KJ, Kaijiang in 2026; 2026BD, Beidou in 2026; BLUP, The best linear unbiased prediction. ^a^ The number of available datasets for the 2CN RIL population in the 2025MC, 2025BD, 2026KJ and 2026BD environments were 171, 156, 184 and 173, respectively. ^b^ *H^2^* was calculated based on the FLC phenotypes across the four test environments.

### 3.2 Correlation analysis between FLC and agronomic, grain-related as well as root system traits

**Figure 2:**
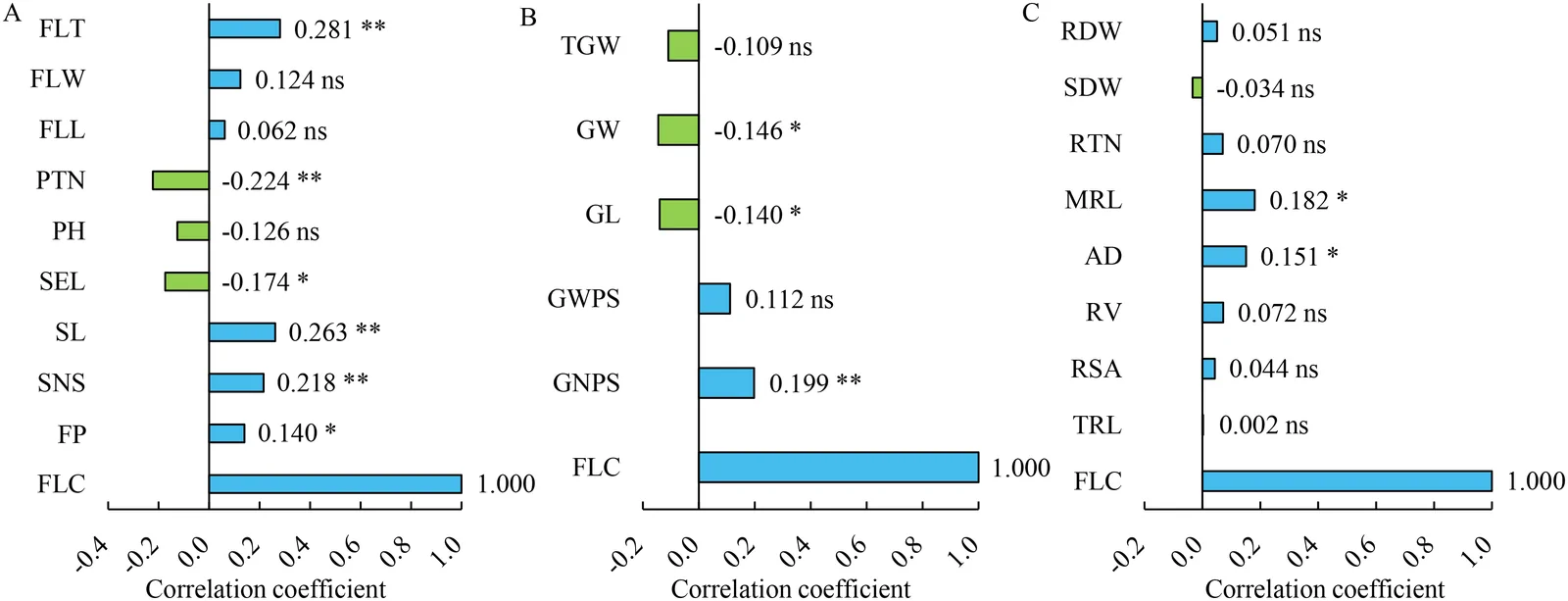
Correlation analysis between flag leaf chlorophyll content (FLC) and agronomic **(A)**, grain-related **(B)** and root system traits **(C)**. * and ** represent significant differences at the 0.05 and 0.01 levels; ns represents no significant differences at the 0.05 levels.

The BLUP datasets were used to systematically evaluate the correlations between FLT and three categories of traits: agronomic traits (FP, SNS, SL, SEL, PH, PTN, FLL, FLW, and FLT), grain-related traits (GNPS, GWPS, GL, GW, and TGW) and root system traits (TRL, RSA, RV, AD, MRL, RTN, SDW, and RDW). For agronomic traits, FLC was significantly positively correlated with FP, SNS, SL and FLT (*P* < 0.05 or *P* < 0.01), whereas significant negative correlations were observed with SEL and PTN (*P* < 0.05 or *P* < 0.01; **Figure 2A**). Regarding grain-related traits, FLC showed a significant positive correlation with GNPS (*P* < 0.01), and significant negative correlations with GL and GW (*P* < 0.05; **Figure 2B**). Among root system traits, only AD and MRL were significantly and positively correlated with FLC (*P* < 0.05; **Figure 2C**). No statistically correlations were identified between FLC and any of the other measured traits (**Figure 2**).

### 3.3 QTL mapping for FLC

**Figure 3:**
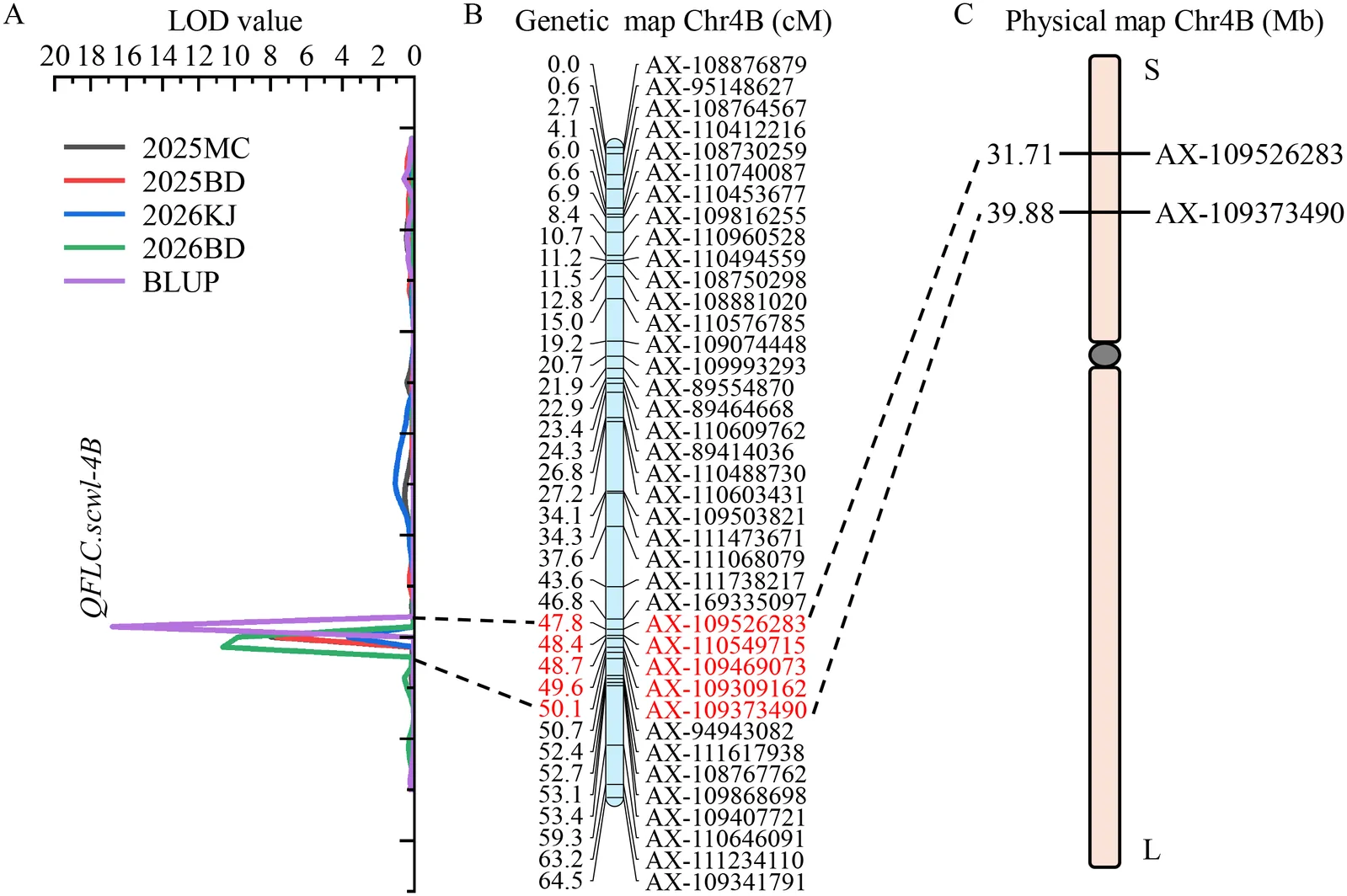
Partial genetic and physical maps of the major QTL *QFLC.scwl-4B*. 2025MC, Micheng in 2025; 2025BD, Beidou in 2025; 2026KJ, Kaijiang in 2026; 2026BD, Beidou in 2026. S and L represent chromosome short and long arm, respectively.

In total, three QTLs controlling FLC were mapped to chromosomes 2A, 3A and 4B (**Table 2**). Among them, *QFLC.scwl-4B* was consistently identified across all four environments and the BLUP dataset, whereas *QFLC.scwl-2A* and *QFLC.scwl-3A* were detected in only a single environment (**Table 2**). Accordingly, *QFLC.scwl-4B* was considered as a major and stable QTL. This locus was located on chromosome arm 4BS, flanked by markers *AX-109526283* and *AX-109373490*, with LOD scores ranging from 3.68 to 16.91 and explaining 9.36%–32.17% of the PVE; (**Table 2**, **Figures 3A, B**). As expected, the positive alleles of *QFLC.scwl-4B* was contributed by parent 20828 (**Table 2**). *QFLC.scwl-2A* and *QFLC.scwl-3A* explained 6.86% and 6.78% of the PVE, respectively (**Table 2**).

**Table 2:** Quantitative trait loci (QTL) for flag leaf chlorophyll content (FLC) identified from different environments in 2CN population.

QTL	Env	Chr	Position (cM)	Left marker	Right marker	LOD	PVE (%)	Add
*QFLC.scwl-2A*	2026BD	2A	85	*AX-110017739*	*AX-109277755*	2.98	6.86	0.70
*QFLC.scwl-3A*	2026KJ	3A	106	*AX-110371883*	*AX-110548249*	2.73	6.78	-0.40
*QFLC.scwl-4B*	BLUP	4B	48	*AX-109526283*	*AX-110549715*	16.91	32.17	0.44
	2025MC		49	*AX-109469073*	*AX-109309162*	7.98	20.24	0.78
	2025BD		49	*AX-109469073*	*AX-109309162*	7.68	19.22	1.14
	2026KJ		49	*AX-109469073*	*AX-109309162*	3.68	9.36	0.48
	2026BD		50	*AX-109309162*	*AX-109373490*	10.65	24.09	1.33

Env, Environment; Chr, Chromosome; LOD, logarithm of odds; PVE, phenotypic variance explained; Add, additive effect of a QTL; 2025MC, Micheng in 2025; BD, Beidou in 2025; 2026KJ, Kaijiang in 2026; 2026BD, Beidou in 2026; BLUP, The best linear unbiased prediction.

Of 15 QTLs identified through multi-environment QTL analysis, three (*QFLC.scwl-4B*, *QFLC.scwl-2A* and *QFLC.scwl-3A*) were identified by both the BIP (**Table 2**) and MET-ADD modules (**Supplementary Table 2**). Notably, only *QFLC.scwl-4B* exhibited high LOD and PVE values, further supporting its designation as a major and stable locus (**Supplementary Table 2**). In addition, eight pairs of epistatic QTLs associated with FLC were identified (**Supplementary Table 3**). Among these, five pairs showed positive additive-by-additive (Add×Add) effect, indicating that the epistatic effects of the parental genotypes were greater than those of recombinant genotypes, whereas the remaining three pairs exhibited negative Add×Add effects. The LOD and PVE values for these epistatic QTLs ranged from 5.04 to 8.35 and from 4.97% to 14.64%, respectively. Interestingly, an epistatic interaction was detected between *QFLC.scwl-2A* and a locus located on chromosome 2A (*AX-110379064*–*AX-109917880*, designated *QFLC.scwl-2A*/2A; **Supplementary Table 3**), with a LOD value of 8.35 and a PVE value of 5.52%.

### 3.4 Validation of the major QTL *QFLC.scwl-4B*

**Figure 4:**
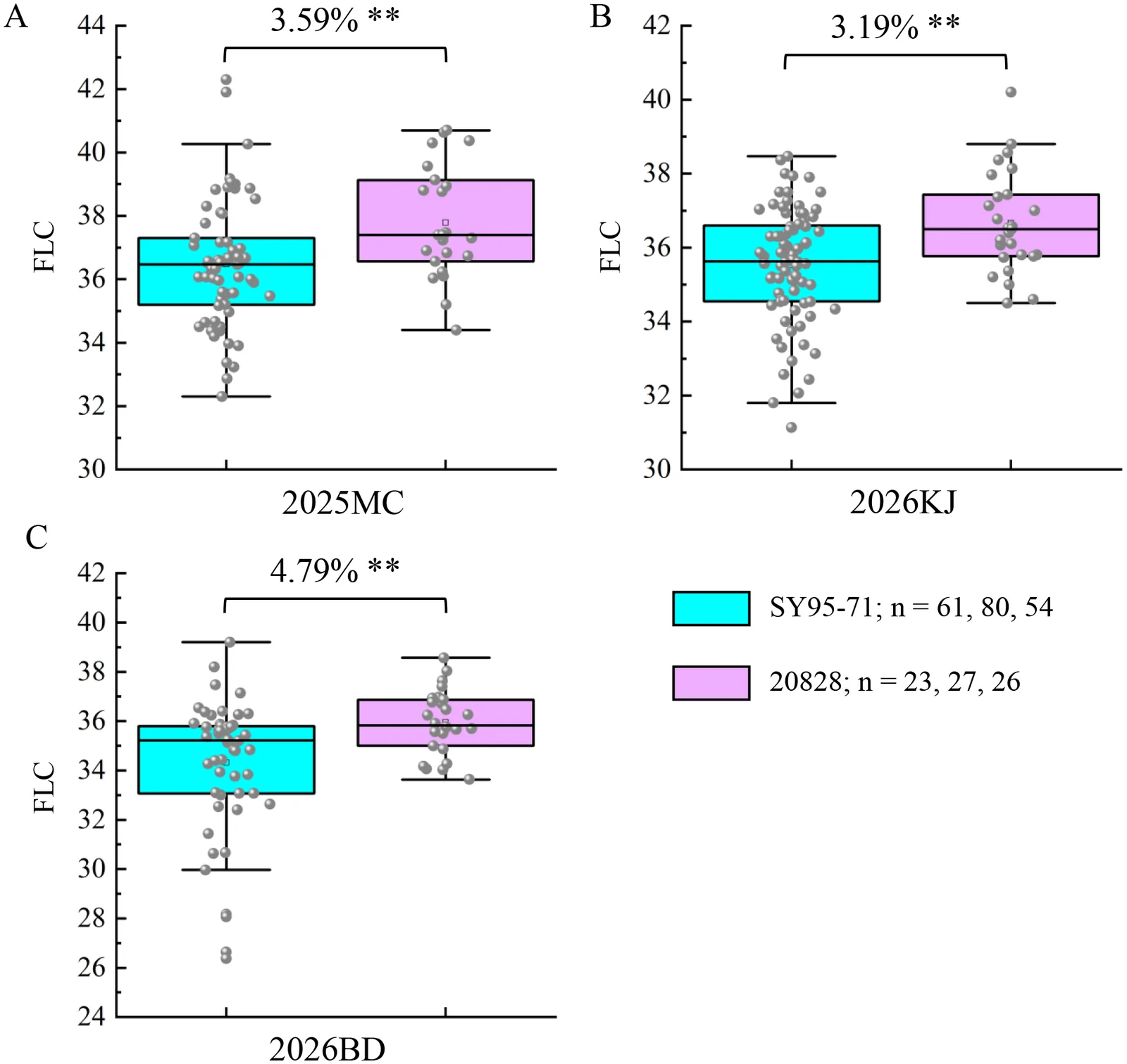
The genetic effects of the major QTL *QFLC.scwl-4B* in 20828/SY95-71 (2SY) population across multiple environments. 2025BD, Beidou in 2025; 2026KJ, Kaijiang in 2026; 2026BD, Beidou in 2026. ** represents significant differences at the 0.01 levels.

The 2SY population was used to assess the genetic effects of *QFLC.scwl-4B* across different environments. Using the markers *AX-109526283* and *AX-109469073* (**Supplementary Table 4**; Liu et al., 2020), the population was divided into two groups: lines carrying the homozygous positive allele from 20828 and those carrying the homozygous allele from SY95-71. In the 2025MC, 2026KJ, and 2026BD environments, lines harboring the positive alleles from 20828 exhibited significantly higher FLC than those carrying alleles from SY95-71 (**Figures 4A–C**). The FLC difference between the two groups ranged from 3.19% to 4.79%, with a mean of 3.86% across the three environments.

### 3.5 Effect of 1BL/1RS translocation on FLC

**Figure 5:**
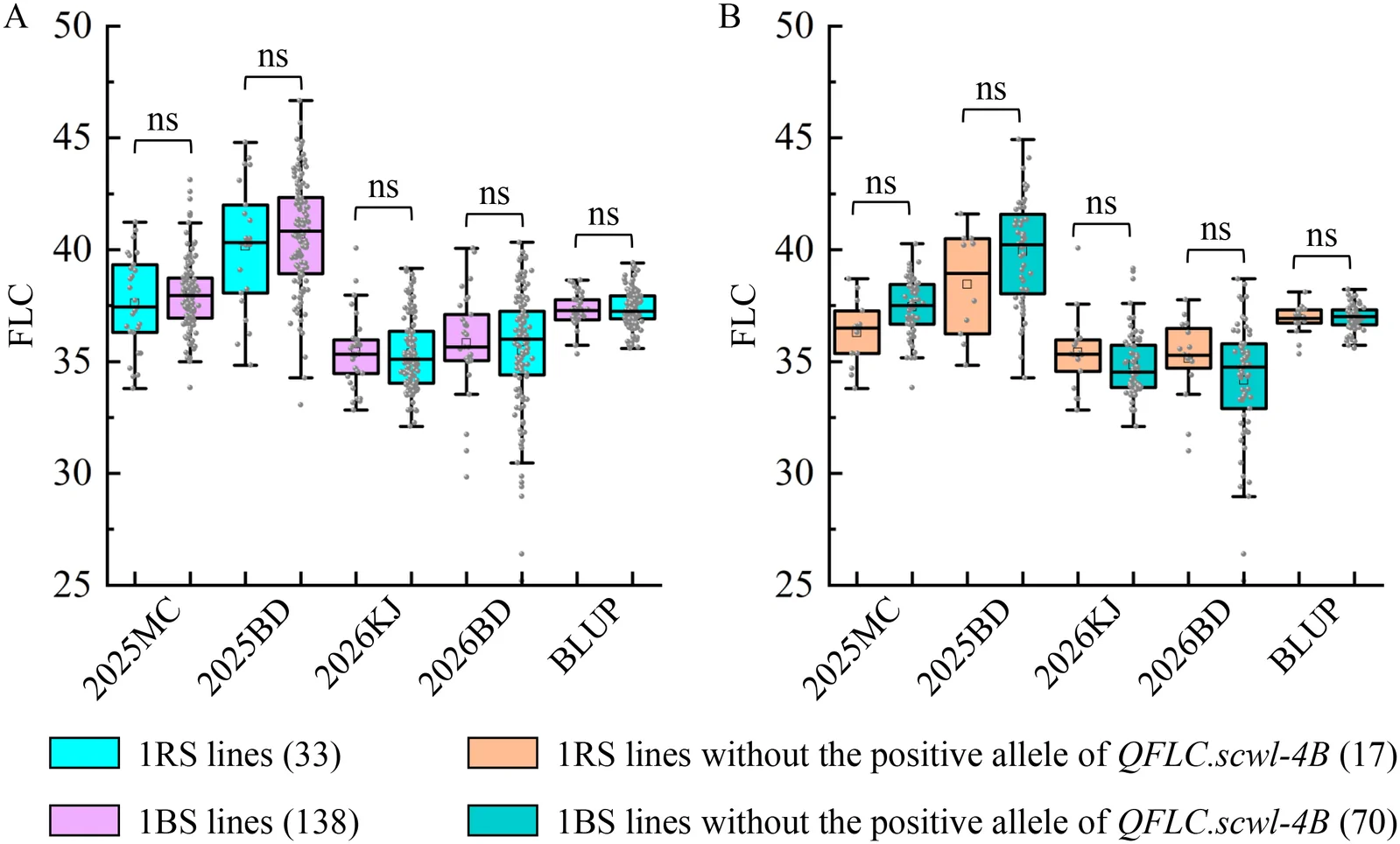
The effect of 1BL/1RS translocation on flag leaf chlorophyll content (FLC). ns represents no significant differences at the 0.05 levels.

No significant difference in FLC was observed between 1BS and 1RS lines across all four environments and the BLUP dataset (**Figure 5A**). To eliminate potential confounding effects of the major QTL, we further compared FLC between 1BS and 1RS lines after excluding those carrying the positive allele of *QFLC.scwl-4B*. In this subset analysis, no significant difference in FLC was detected between the two groups (**Figure 5B**). Taken together, these findings suggest that the 1BL/1RS translocation does not affect FLC.

### 3.6 Effects of *QFLC.scwl-4B* on agronomic, grain-related and root system traits

**Figure 6:**
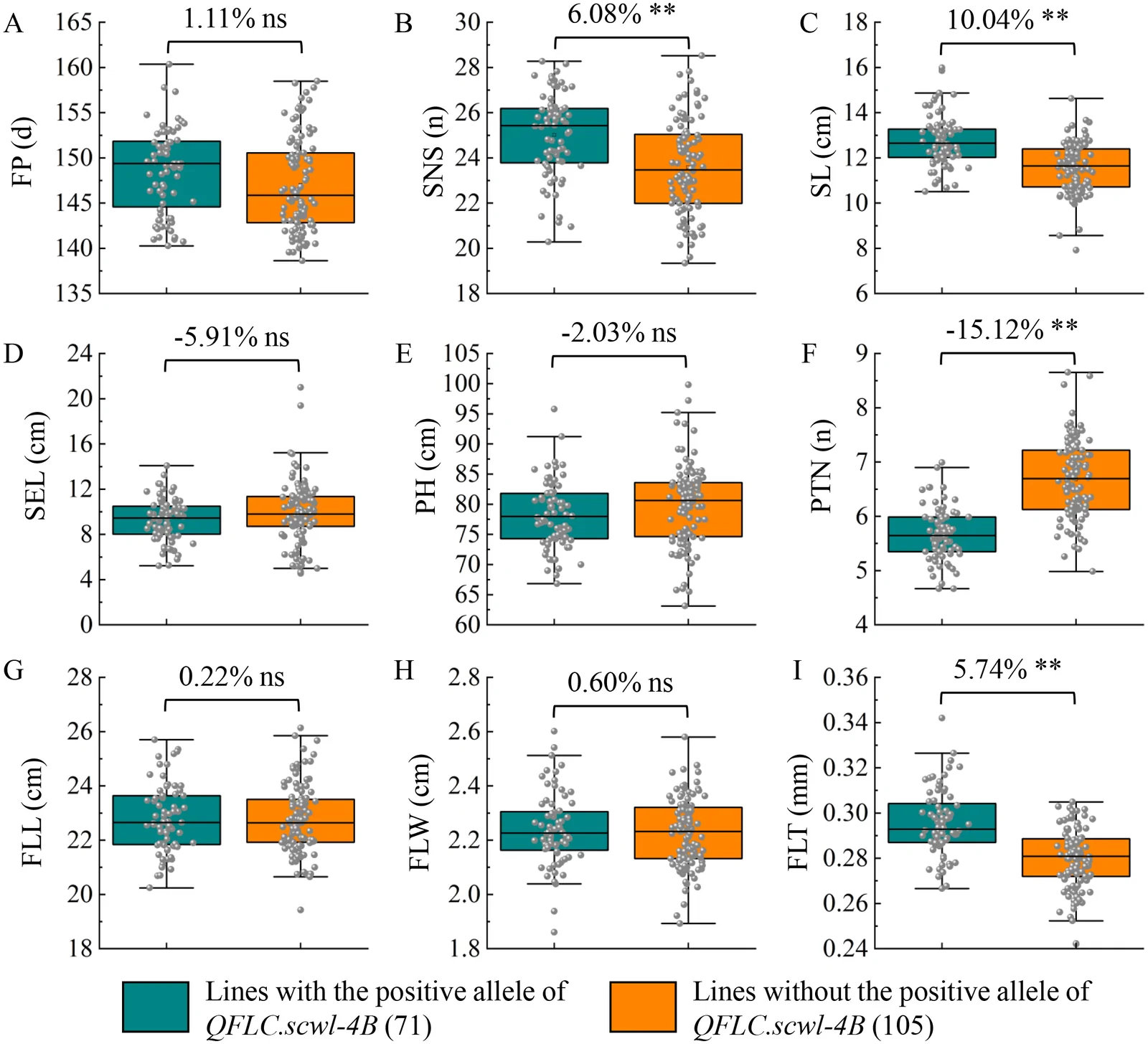
The effect of *QFLC.scwl-4B* on agronomic traits. ** represents significant differences at the 0.01 levels; ns represents no significant differences at the 0.05 levels.

The effects of *QFLC.scwl-4B* on agronomic, grain-related, and root system traits were further analyzed. For agronomic traits, lines carrying the positive allele exhibited a significant decrease of 15.12% in PTN (*P* < 0.01; **Figure 6F**), whereas SNS, SL and FLT were significantly increased by 6.08%, 10.04%, and 5.74%, respectively (*P* < 0.01; **Figures 6B, C, I**). Regarding grain-related traits, these lines exhibited significant increase of 3.01% (*P* < 0.05) in GNPS and 2.03% (*P* < 0.01) in GWPS (**Supplementary Figures 2A, B**). For root system traits, they showed significant increases in RSA (4.40%, *P* < 0.01), RV (5.08%, *P* < 0.01), AD (0.97%, *P* < 0.01), MRL (4.24%, *P* < 0.01), and RDW (3.15%, *P* < 0.05; **Supplementary Figures 3B–E**, H). No significant differences were observed between the two groups for the remaining 11 traits (**Figure 6A, D, E, G, H**; **Supplementary Figure 2C–E**; **Supplementary Figure 3A, F, G**). In addition, co-localization was observed between QTL for FLC and those governing SNS, PH, FLT and PTN in the 2CN population (**Supplementary Table 5**). Collectively, these results indicate that *QFLC.scwl-4B* is pleiotropic, acting to upregulate FLC, SNS and FLT while downregulating PTN.

## 4 Discussion

### 4.1 Comparison of *QFLC.scwl-4B* to other loci governing FLC

In this study, *QFLC.scwl-4B* was physically mapped between markers *AX-109526283* (31.71 Mb) and *AX-109373490* (39.88 Mb) on chromosome arm 4BS (**Table 2**, **Figure 3C**). To date, few QTLs for FLC have been reported on chromosome 4B in wheat (**Supplementary Table 6**). Among the previously reported loci, *qCHN-4B*, *QChl-4B.1*, *Qspad.acs-4B.1* and *Qspad.acs-4B.3* were all located on chromosome arm 4BL, far from the 4BS position of *QFLC.scwl-4B* located on 4BS (Cao et al., 2004; Yang et al., 2016a, Yang et al., 2016b). *Qspad.acs-4B.2* was positioned at 8.39 Mb on 4BS, showing no physical overlap with *QFLC.scwl-4B* (Yang et al., 2016b). Additionally, *QSPAD-10D.xjau-4B* (174.89–176.06 Mb), *QSPAD-14D.xjau-4B* (174.89–262.95 Mb), and *QSPAD-18D.xjau-4B* (174.89–262.95 Mb), also located on 4BS, were considerably distant from *QFLC.scwl-4B* (Chen et al., 2024). Notably, *Qchl.saw-4B* was mapped to the 31.50–53.56 Mb interval of 4BS (Yang et al., 2023), which overlaps with the region of *QFLC.scwl-4B* (31.71–39.88 Mb), suggesting that these two loci may be allelic. However, the aforementioned QTL for FLC were each detected in a single environment and classified as minor QTLs (**Supplementary Table 6**). In contrast, *QFLC.scwl-4B* was consistently detected across all four test environments and the BLUP dataset, with LOD scores ranging from 3.68 to 16.91 and PVE values from 9.36% to 32.17% (**Table 2**). Multi-environment QTL analysis further confirmed its stability (**Supplementary Table 2**). Collectively, these results demonstrate that *QFLC.scwl-4B* is a major and environmentally stable QTL governing FLC.

Moreover, the physical interval of *QFLC.scwl-4B* (31.71–39.88 Mb) encompasses previously identified QTLs for TGW, including *QTKW.caas-4BS* (31.71–35.23 Mb; Xu et al., 2019), *QTgw.cau-4B-1.1* (22.43–40.94 Mb; Wen et al., 2022), and *QTgw.caas-4B* (25.69–34.90 Mb; Li et al., 2024), indicating that this genomic region serves as a key regulator of multiple agronomically important traits. Therefore, *QFLC.scwl-4B* not only provides a robust locus for FLC but also represents a promising target for dissecting the genetic basis of yield-related traits, thereby facilitating marker-assisted selection in wheat breeding.

### 4.2 Factors underlying the limited number of FLC QTL detected

In this study, only three QTLs were identified, of which only one was major and stable (**Table 2**). This outcome is not exceptional, as similarly low numbers of FLC QTLs have been reported previously (Hassan et al., 2018; Tahmasebi et al., 2017; Wang et al., 2022). FLC is a typical complex quantitative trait, and the detection of its underlying QTL is subject to multiple influencing factors (Watson et al., 2024). One critical factor is population size. The mapping population used in this study comprised approximately 200 lines, which may have constrained the statistical power, particularly for detecting minor QTL. It is therefore plausible that additional minor QTL could be uncovered by employing a larger population. A second limiting factor is marker density and mapping resolution. Although the marker density in this study was adequate for initial QTL detection, it may have been sufficient to fully resolve all QTL, potentially leading to an underestimation of the total number of QTLs identified.

Furthermore, environmental variation, including moisture availability, soil nutrient, temperature, and light, is known to substantially influence the expression and detectability of QTLs underlying physiological traits such as FLC, which likely contributed to the limited number of stable QTLs observed in this study. Collectively, the small number of QTLs detected here is likely attributable to the combined effects of population size, marker density and environmental factor. Despite these limitations, the identification of a single stable and major QTL represents a valuable genetic resource, offering a solid foundation for further functional characterization and for potential application in marker-assisted breeding.

### 4.3 Predicted genes in the interval of *QFLC.scwl-4B*

Based on the physical interval of *QFLC.scwl-4B* in the CS v2.1 genome (31.71–39.88 Mb), 65 high confidence genes were annotated (**Supplementary Table 7**). Expression analysis revealed that 43 of these genes were expressed in leaves, while the remaining 22 showed negligible expression (**Supplementary Table 7**). Notably, four genes (*TraesCS4B03G0095100*, *TraesCS4B03G0097700*, *TraesCS4B03G0098300* and *TraesCS4B03G0103100*) exhibited notably higher expression levels in leaves than in other tissues (**Supplementary Figure 4**). Among them, *TraesCS4B03G0095100*, *TraesCS4B03G0097700*, and *TraesCS4B03G0103100* encode a peptidylprolyl isomerase, an AT-rich interactive domain protein, and a NADH-ubiquinone oxidoreductase 75 kDa subunit, mitochondrial, respectively (**Supplementary Table 7**). However, no previous studies have reported the involvement of these three genes in chlorophyll synthesis. By contrast, *TraesCS4B03G0098300* encodes a CONSTANS-like zinc finger protein, and is an ortholog of the *BBX* family genes characterized in diverse plant species, including *Arabidopsis* and chrysanthemum. BBX proteins have been implicated in plant growth and development (Cao et al., 2023). In particular, *CmBBX22* has been shown to repress chlorophyll degradation- and senescence-related genes expression (e.g., *ABF4*, *SAG29*, and *NYE1/2*), thereby delaying senescence in *Arabidopsis* (Liu et al., 2019a, Liu et al., 2019b). Further, sequence analysis revealed that the CDS of *TraesCS4B03G0098300* showed no sequence differences between the parents 20828 and CN16 (**Supplementary Figure 5A**), while its promoter sequence contained a single nucleotide polymorphism (G/C) between the two parents (**Supplementary Figure 5B**). Collectively, these observations suggest that *TraesCS4B03G0098300* is likely involved in chlorophyll synthesis and may serve as a promising candidate for fine mapping and cloning of *QFLC.scwl-4B*.

### 4.4 Correlation of FLC with other traits and its breeding significance

Correlation analysis based on the BLUP datasets revealed that FLC was significantly positively correlated with SNS, SL, FLT and GNPS, whereas it was significantly negatively correlated with SEL, PTN, GL, and GW (**Figures 2A, B**). These correlations are consistent with the pleiotropic effects of *QFLC.scwl-4B*, suggesting that this locus largely underpins phenotypic associations observed among the above agronomic traits. Notably, the positive correlation between FLC and SNS is particular interest. Although FLC and SNS regulate flag leaf physiological status and spike development, respectively, their concurrent improvement may enhance canopy photosynthetic capacity and increase yield potential (Du et al., 2022; Ma et al., 2019a). In contrast, the negative correlation between FLC and PTN points to a typical genetic trade-off, as genotypes selected for higher FLC tend to produce fewer PTN, which may ultimately limit grain yield formation (Liu et al., 2018, Liu et al., 2020). Regarding root system traits, only AD and MRL exhibited significant positive correlations with FLC (**Figure 2C**), suggesting a degree of synergistic regulation between FLC and root system architecture. This observation highlights the potential for breeding wheat varieties with improved resource capture by selecting for higher FLC and deeper root systems, thereby enhancing both above- and below-ground resource acquisition (Alemu et al., 2021; Chen et al., 2022). Collectively, these correlation patterns provide valuable guidance for designing breeding strategies that balance flag leaf improvement, tiller maintenance, and root system optimization to achieve sustainable yield gains under diverse production conditions.

## 5 Conclusion

In this study, a major, stable and validated QTL for FLC, *QFLC.scwl-4B*, was identified using an RIL population. This QTL was consistently detected across multiple environments, accounting for up to 32.17% of the PVE. In addition, genetic correlations between FLC and agronomic, grain-related, and root system traits were systematically evaluated. *QFLC.scwl-4B* co-localized with QTLs for SNS, PH, FLT and PTN in the 2CN population, supporting its pleiotropic. Furthermore, the 1BL/1RS translocation was found to exert no significant effect on FLC. Collectively, these findings establish a foundation for fine mapping of *QFLC.scwl-4B* and for its eventual applications in marker-assisted breeding.

## Data Availability

The datasets presented in this study can be found in online repositories. The names of the repository/repositories and accession number(s) can be found in the article/**Supplementary Material**.

## References

[B1] Alemu A. Feyissa T. Maccaferri M. Sciara G. Tuberosa R. Ammar K. . (2021). Genome-wide association analysis unveils novel QTLs for seminal root system architecture traits in Ethiopian durum wheat. BMC Genomics 22, 20. doi: 10.1186/s12864-020-07320-4 33407083 PMC7789649

[B2] Avenson T. J. Cruz J. A. Kanazawa A. Kramer D. M. (2005). Regulating the proton budget of higher plant photosynthesis. Proc. Natl. Acad. Sci. U.S.A. 102, 9709–9713. doi: 10.1073/pnas.0503952102 15972806 PMC1172270

[B3] Boden S. A. McIntosh R. A. Uauy C. Krattinger S. G. Dubcovsky J. Rogers W. J. . (2023). Updated guidelines for gene nomenclature in wheat. Theor. Appl. Genet. 36, 72. doi: 10.1007/s00122-023-04253-w 36952017 PMC10036449

[B4] Cao J. Yuan J. L. Zhang Y. L. Chen C. Zhang B. H. Shi X. M. . (2023). Multi-layered roles of BBX proteins in plant growth and development. Stress Biol. 3, 1. doi: 10.1007/s44154-022-00080-z 37676379 PMC10442040

[B5] Cao W. D. Jia J. Z. Jin J. Y. (2004). Identification and interaction analysis of QTL for chlorophyll content in wheat seedlings. J. Plant Nutr. Fertil. 10, 473–478. doi: 10.11674/zwyf.2004.050542636401. abstract in English.

[B6] Chang C. Lu J. Zhang H. P. Ma C. X. Sun G. (2015). Copy number variation of cytokinin oxidase gene *Tackx4* associated with grain weight and chlorophyll content of flag leaf in common wheat. PLoS One 10, e0145970. doi: 10.1371/journal.pone.0145970 26714276 PMC4699907

[B7] Chaudhary S. Jha U. C. Paul P. J. Prasad P. V. V. Sharma K. D. Kumar S. . (2022). Assessing the heat sensitivity of Urdbean (*Vigna mungo* L. Hepper) genotypes involving physiological, reproductive and yield traits under field and controlled environment. Front. Plant Sci. 13, 1042999. doi: 10.3389/fpls.2022.1042999 36507460 PMC9733429

[B8] Chen C. Chen Y. K. Wang W. Ren Y. Zhang H. Y. Chen H. B. . (2024). QTL mapping of stay-green-related traits in wheat under drought condition. Acta Agron. Sin. 50, 2684–2698. doi: 10.3724/SP.J.1006.2024.41022. abstract in English.

[B9] Chen H. X. Wei J. T. Tian R. Zeng Z. Y. Tang H. P. Liu Y. L. . (2022). A major quantitative trait locus for wheat total root length associated with precipitation distribution. Front. Plant Sci. 13, 995183. doi: 10.3389/fpls.2022.995183 36092437 PMC9451531

[B10] Chen H. Y. Zhang S. T. Chang J. B. Wei H. R. Li H. C. Li C. Y. . (2025). Foliar application of 24-epibrassinolide enhances leaf nicotine content under low temperature conditions during the mature stage of flue-cured tobacco by regulating cold stress tolerance. BMC Plant Biol. 25, 77. doi: 10.1186/s12870-025-06080-1 39828684 PMC11744823

[B11] Cui S. Liu S. R. Zhang Y. Zhang X. Y. Feng G. Z. Wang S. J. . (2026). Photosynthesis-driven yield improvement in maize under sulfur application: role of young ear development and grain filling. BMC Plant Biol. 26, 371. doi: 10.1186/s12870-026-08248-9 41688932 PMC12927233

[B12] Du B. B. Wu J. Islam M. S. Sun C. Y. Lu B. W. Wei P. P. . (2022). Genome-wide meta-analysis of QTL for morphological related traits of flag leaf in bread wheat. PLoS One 17, e0276602. doi: 10.1371/journal.pone.0276602 36279291 PMC9591062

[B13] Feng K. H. Qi Y. H. Ye Z. X. Li T. Jiao G. Y. Zhang C. X. . (2025). Diversity and evolution analysis of RNA viruses in three wheat aphid species. BMC Genomics 26, 353. doi: 10.1186/s12864-025-11512-1 40197145 PMC11978097

[B14] Han Z. Y. Yu L. Q. Li X. Wang S. R. Ding K. Zhao B. Q. . (2026). Successful modification of a commercial wheat variety, lunxuan 13, for pre-harvest sprouting resistance through editing of the *TaQsd1* gene. Plants 15, 1322. doi: 10.3390/plants15091322 42122816 PMC13165390

[B15] Hassan F. S. C. Solouki M. Fakheri B. A. Nezhad N. M. Masoudi B. (2018). Mapping QTLs for physiological and biochemical traits related to grain yield under control and terminal heat stress conditions in bread wheat (*Triticum aestivum* L.). Physiol. Mol. Biol. Plants 24, 1231–1243. doi: 10.1007/s12298-018-0590-8 30425437 PMC6214426

[B16] Ibrahim A. R. Moursy M. E. S. Abdelghany A. M. Lamlom S. F. (2025). Impact of cultivars, nitrogen rates, and sugar beet processing by-product on wheat growth and yield. BMC Plant Biol. 25, 1567. doi: 10.1186/s12870-025-07560-0 41239221 PMC12616930

[B17] Ilyas M. Ilyas N. Arshad M. Kazi A. Kazi A. Waheed A. (2014). QTL mapping of wheat doubled haploids for chlorophyll content and chlorophyll fluorescence kinetics under drought stress imposed at anthesis stage. Pak. J. Bot. 46, 1889–1897. Available online at: https://www.researchgate.net/publication/27262127.

[B18] Li Y. H. Hu J. H. Qu Y. F. Qiu D. Lin H. L. Du J. Y. . (2024). Alleles on locus chromosome 4B from different parents confer tiller number and the yield-associated traits in wheat. BMC Plant Biol. 24, 454. doi: 10.1186/s12870-024-05079-4 38789943 PMC11127307

[B19] Li M. M. Li B. B. Guo G. H. Chen Y. X. Xie J. Z. Lu P. . (2018). Mapping a leaf senescence gene *els1* by BSR-Seq in common wheat. Crop J. 6, 236–243. doi: 10.1016/j.cj.2018.01.00442574925

[B20] Li C. Ma J. Liu H. Ding P. Y. Yang C. C. Zhang H. . (2019). Detection of QTLs for spike length and plant height in wheat based on 55K SNP array. J. Triticeae Crops 39, 1284–1292. doi: 10.7606/j.issn.1009-1041.2019.11.03. abstract in English.

[B21] Li T. Ma J. Zou Y. Y. Chen G. D. Ding P. Y. Zhang H. . (2020b). Quantitative trait loci for seeding root traits and the relationships between root and agronomic traits in common wheat. Genome 63, 27–36. doi: 10.1139/gen-2019-0116 31580743

[B22] Li C. Tang H. Luo W. Zhang X. M. Mu Y. Deng M. . (2020a). A novel, validated, and plant height-independent QTL for spike extension length is associated with yield-related traits in wheat. Theor. Appl. Genet. 133, 3381–3393. doi: 10.1007/s00122-020-03675-0 32870326

[B23] Li H. W. Tong Y. P. Li B. Jing R. L. Lu C. M. Li Z. S. (2010). Genetic analysis of tolerance to photo-oxidative stress induced by high light in winter wheat (*Triticum aestivum* L.). J. Genet. Genom. 37, 399–412. doi: 10.1016/S1673-8527(09)60058-8 20621022

[B24] Liu Y. N. Chen H. Qi P. Zhang Z. X. Guan Z. Y. Fang W. M. . (2019b). The heterologous expression of *CmBBX22* delays leaf senescence and improves drought tolerance in *Arabidopsis*. Plant Cell Rep. 38, 15–24. doi: 10.1007/s00299-018-2345-y 30238422

[B25] Liu X. Li R. Dai Y. Q. Yuan L. Sun Q. H. Zhang S. Z. . (2019a). A B-box zinc finger protein, *MdBBX10*, enhanced salt and drought stresses tolerance in *Arabidopsis*. Plant Mol. Biol. 99, 437–447. doi: 10.1007/s11103-019-00828-8 30712230

[B26] Liu J. J. Luo W. Qin N. N. Ding P. Y. Zhang H. Yang C. C. . (2018). A 55 K SNP array-based genetic map and its utilization in QTL mapping for productive tiller number in common wheat. Theor. Appl. Genet. 131, 2439–2450. doi: 10.1007/s00122-018-3164-9 30109392

[B27] Liu J. J. Tang H. P. Qu X. R. Liu H. Li C. Tu Y. . (2020). A novel, major, and validated QTL for the effective tiller number located on chromosome arm 1BL in bread wheat. Plant Mol. Biol. 104, 173–185. doi: 10.1007/s11103-020-01035-6 32734417

[B28] Liu J. J. Tao L. Qin N. N. Liu J. T. Wang D. F. Liu X. R. . (2026). Novel loci for flag leaf thickness with breeding potential: genetic dissection and candidate genes prediction. Front. Plant Sci. 10, 1906045. doi: 10.3389/fpls.2026.1906045 42591623. . PMC13462394

[B29] Liu J. J. Wang T. Z. Lan Y. X. Zhang Z. Y. You J. N. Wu L. . (2025). Fine-mapping and candidate gene identification for *QPtn.sau-4B* showing potential in increasing productive tiller number and yield in wheat. Crop J. 13, 480–489. doi: 10.1016/j.cj.2025.01.01442574925

[B30] Ma J. Ding P. Y. Liu J. J. Li T. Zou Y. Y. Habib A. . (2019a). Identification and validation of a major and stably expressed QTL for spikelet number per spike in bread wheat. Theor. Appl. Genet. 132, 3155–3167. doi: 10.1007/s00122-019-03415-z 31435704

[B31] Ma J. Qin N. N. Cai B. Chen G. Y. Ding P. Y. Zhang H. . (2019b). Identification and validation of a novel major QTL for all-stage stripe rust resistance on 1BL in the winter wheat line 20828. Theor. Appl. Genet. 132, 1363–1373. doi: 10.1007/s00122-019-03283-7 30680420

[B32] Ma J. Tu Y. Zhu J. Luo W. Liu H. Li C. . (2020). Flag leaf size and posture of bread wheat: genetic dissection, QTL validation and their relationships with yield-related traits. Theor. Appl. Genet. 133, 297–315. doi: 10.1007/s00122-019-03458-2 31628527

[B33] Ma J. Zhang H. Li S. Q. Zou Y. Y. Li T. Liu J. J. . (2019c). Identification of quantitative trait loci for kernel traits in a wheat cultivar Chuannong16. BMC Genet. 20, 77. doi: 10.1186/s12863-019-0782-4 31619163 PMC6796374

[B34] Meng L. Li H. Zhang L. Y. Wang J. K. (2015). QTL IciMapping: Integrated software for genetic linkage map construction and quantitative trait locus mapping in biparental populations. Crop J. 3, 269–283. doi: 10.1016/j.cj.2015.01.00142574925

[B35] Netto A. T. Campostrini E. Oliveira J. G. D. Bressan-Smith R. E. (2005). Photosynthetic pigments, nitrogen, chlorophyll a fluorescence and SPAD-502 readings in coffee leaves. Sci. Hortic. 104, 199–213. doi: 10.1016/j.scienta.2004.08.01342574925

[B36] Qi W. L. Tang Y. Zhu W. Li D. Y. Diao C. D. Xu L. L. . (2016). Molecular cytogenetic characterization of a new wheat-rye 1BL·1RS translocation line expressing superior stripe rust resistance and enhanced grain yield. Planta 244, 405–416. doi: 10.1007/s00425-016-2517-3 27084678

[B37] Ren T. H. Fan T. Chen S. L. Chen Y. Y. Ou X. Jiang Q. . (2022). Identification and validation of quantitative trait loci for the functional stay green trait in common wheat (*Triticum aestivum* L.) via high-density SNP-based genotyping. Theor. Appl. Genet. 135, 1429–1441. doi: 10.1007/s00122-022-04044-9 35138422

[B38] Shi S. K. Azam F. I. Li H. H. Chang X. P. Li B. Y. Jing R. L. (2017). Mapping QTL for stay-green and agronomic traits in wheat under diverse water regimes. Euphytica 213, 246. doi: 10.1007/s10681-017-2002-530311153

[B39] Tahmasebi S. Heidari B. Pakniyat H. McIntyre C. L. (2017). Mapping QTLs associated with agronomic and physiological traits under terminal drought and heat stress conditions in wheat (*Triticum aestivum* L.). Genome 60, 26–45. doi: 10.1139/gen-2016-0017 27996306

[B40] Wang W. Gao X. Cheng Y. K. Ren Y. Zhang Z. H. Wang R. . (2022). QTL mapping of leaf area index and chlorophyll content based on UAV remote sensing in wheat. Agriculture 12, 595. doi: 10.3390/agriculture1205059530654563

[B41] Wang Z. L. Shen M. M. Yang H. Du X. F. Han K. N. Wu H. P. . (2025). Combining QTL mapping and RNA-Seq reveals candidate genes controlling flag leaf width in foxtail millet. BMC Plant Biol. 25, 1365. doi: 10.1186/s12870-025-07358-0 41073899 PMC12512300

[B42] Wang H. Y. Wang S. G. Chang X. P. Hao C. Y. Sun D. Z. Jing R. L. (2019). Identification of *TaPPH-7A* haplotypes and development of a molecular marker associated with important agronomic traits in common wheat. BMC Plant Biol. 19, 296. doi: 10.1186/s12870-019-1901-0 31286893 PMC6615193

[B43] Wang N. Xie Y. Z. Li Y. Z. Wu S. X. Li S. X. Guo Y. . (2020). High-resolution mapping of the novel early leaf senescence gene *Els2* in common wheat. Plants 9, 698. doi: 10.3390/plants9060698 32486195 PMC7355531

[B44] Watson A. E. Guitton B. Soriano A. Rivallan R. Vignes H. Farrera I. . (2024). Target enrichment sequencing coupled with GWAS identifies *MdPRX10* as a candidate gene in the control of budbreak in apple. Front. Plant Sci. 15, 1352757. doi: 10.3389/fpls.2024.1352757 38455730 PMC10918860

[B45] Wen S. Z. Zhang M. H. Tu K. L. Fan C. F. Tian S. Bi C. . (2022). A major quantitative trait loci cluster controlling three components of yield and plant height identified on chromosome 4B of common wheat. Front. Plant Sci. 12, 799520. doi: 10.3389/fpls.2021.799520 35087558 PMC8786729

[B46] Xiong D. L. Chen J. Yu T. T. Gao W. L. Ling X. X. Li Y. . (2015). SPAD-based leaf nitrogen estimation is impacted by environmental factors and crop leaf characteristics. Sci. Rep. 5, 13389. doi: 10.1038/srep13389 26303807 PMC4548214

[B47] Xu D. G. Wen W. E. Fu L. P. Li F. J. Li J. H. Xie L. . (2019). Genetic dissection of a major QTL for kernel weight spanning the *Rht-B1* locus in bread wheat. Theor. Appl. Genet. 132, 3191–3200. doi: 10.1007/s00122-019-03418-w 31515582

[B48] Yang B. Chen N. Dang Y. F. Wang Y. Z. Wen H. W. Zheng J. . (2022). Identification and validation of quantitative trait loci for chlorophyll content of flag leaf in wheat under different phosphorus treatments. Front. Plant Sci. 13, 1019012. doi: 10.3389/fpls.2022.1019012 36466250 PMC9714299

[B49] Yang D. L. Jing R. L. Chang X. P. Li W. (2007). Quantitative trait loci mapping for chlorophyll fluorescence and associated traits in wheat (*Triticum aestivum*). J. Integr. Plant Biol. 49, 646–654. doi: 10.1111/j.1744-7909.2007.0044.x40046247

[B50] Yang D. L. Li M. F. Liu Y. Chang L. Cheng H. B. Chen J. J. . (2016b). Identification of quantitative trait loci and water environmental interactions for developmental behaviors of leaf greenness in wheat. Front. Plant Sci. 7, 273. doi: 10.3389/fpls.2016.00273 27014298 PMC4782216

[B51] Yang B. Qiao L. Zhao J. J. Wu B. B. Wen H. W. Zhang S. W. . (2023). QTL mapping and validation of chlorophyll content of flag leaves in wheat (*Triticum aestivum* L.). Acta Agron. Sin. 49, 744–754. doi: 10.3724/SP.J.1006.2023.21018. abstract in English.

[B52] Yang B. Yan X. Wang H. Y. Li X. Y. Ma H. Wang S. G. . (2016a). Dynamic QTL analysis of chlorophyll content during grain filling stage in winter wheat (*Triticum aestivum* L.). Rom. Agric. Res. 33, 77–85. Available online at: https://www.researchgate.net/publication/303148421.

[B53] Zhang K. P. Fang Z. J. Liang Y. Tian J. C. (2009a). Genetic dissection of chlorophyll content at different growth stages in common wheat. J. Genet. 88, 183–189. doi: 10.1007/s12041-009-0026-x 19700856

[B54] Zhang K. Zhang Y. Chen G. Tian J. (2009b). Genetic analysis of grain yield and leaf chlorophyll content in common wheat. Cereal Res. Commun. 37, 499–511. doi: 10.1556/crc.37.2009.4.3

[B55] Zhu T. T. Wang L. Rimbert H. Rodriguez J. C. Deal K. R. De Oliveira R. . (2021). Optical maps refine the bread wheat *Triticum aestivum* cv. Chinese Spring genome assembly. Plant J. 107, 303–314. doi: 10.1111/tpj.15289 33893684 PMC8360199

[B56] Zhu X. F. Zhang H. P. Hu M. J. Wu Z. Y. Jiang H. Cao J. J. . (2016). Cloning and characterization of *Tabas1-B1* gene associated with flag leaf chlorophyll content and thousand-grain weight and development of a gene-specific marker in wheat. Mol. Breed. 36, 142. doi: 10.1007/s11032-016-0563-y30311153

