# Supplementary material for: Flag leaf chlorophyll content of wheat: genetic dissection, candidate genes prediction and its relationship with other agronomic traits
Source: Front Plant Sci. 2026 Sep 4;17:1938691. doi: 10.3389/fpls.2026.1938691 (PMC13586006; doi:10.3389/fpls.2026.1938691)
Supplement: Supplementary file 1 [file DataSheet1.docx]

**Supplementary Figure 1** Frequency distribution of flag leaf chlorophyll content (FLC) in the 2CN population. 2025MC, Micheng in 2025; BD, Beidou in 2025; 2026KJ, Kaijiang in 2026; 2026BD, Beidou in 2026; BLUP, The best linear unbiased prediction.

**Supplementary Figure 2** The effect of *QFLC.scwl-4B* on grain-related traits. * and ** represent significant differences at the 0.05 and 0.01 levels; ns represents no significant differences at the 0.05 levels.

**Supplementary Figure 3** The effect of *QFLC.scwl-4B* on root system traits. * and ** represent significant differences at the 0.05 and 0.01 levels; ns represents no significant differences at the 0.05 levels.

**Supplementary Figure 4** Expression patterns of four putative candidate genes for *QFLC.scwl-4B*.

**Supplementary Figure 5** Analysis of the coding sequence (A) and promoter sequence (B) of *TraesCS4B03G0098300.*
